# When multimodal fusion fails: contrastive alignment as a necessary stabilizer for TCR–peptide binding prediction

**DOI:** 10.1093/bioinformatics/btag573

**Published:** 2026-08-06

**Authors:** Cong Qi, Wenbo Wang, Hanzhang Fang, Zhi Wei

**Affiliations:** Department of Computer Science, New Jersey Institute of Technology, Newark, NJ 07102, United States; Department of Computer Science, Hamilton College, Clinton, NY 13323, United States; Department of Computer Science, New Jersey Institute of Technology, Newark, NJ 07102, United States; Department of Computer Science, New Jersey Institute of Technology, Newark, NJ 07102, United States

## Abstract

**Motivation:**

Multimodal learning is often assumed to improve predictive performance by combining complementary views, yet in biological applications auxiliary modalities are frequently imperfect, incomplete, or derived from upstream predictors and heuristics. We study this issue in TCR–peptide binding prediction, where sequence embeddings from pretrained protein language models are strong and transferable, but structure-derived residue graphs must be built from predicted folds and discretized contacts. These structural views can therefore be noisy, inconsistent across proteins, and sensitive to modeling choices, making them difficult to optimize jointly with sequence features. In this setting, naive sequence + graph fusion can destabilize training and degrade generalization, falling below a sequence-only baseline when supervision is scarce or contacts are noisy. This motivates a practical goal: use imperfect structural information when it helps, without sacrificing stability when it does not.

**Results:**

We introduce TRACE, a lightweight framework that encodes each entity (TCR and peptide) with parallel sequence (frozen ESM-2) and residue-graph (GNN) towers, then applies CLIP-style intra-entity contrastive alignment before interaction modeling. The alignment encourages modality-consistent representations for the same biological entity, preventing noisy graph signals from dominating fusion. We evaluate under a leakage-controlled TCHard RN protocol with pair-disjoint splits, training-only model selection, and exclusion of negative-sampling metadata that otherwise trivially inflates AUROC. In this setting the task is near chance for all methods, and we do not claim state-of-the-art accuracy; our contribution is the failure-mode analysis, the alignment stabilizer, and the audited protocol itself. Among matched baselines TRACE attains the best mean AUROC (0.578±0.033 over five folds), and an ablation shows that intra-entity alignment acts as a *stabilizer*: it gives a small but consistent full-data gain (better on 4 of 5 folds), stays robust under substantial graph-edge corruption, and prevents collapse toward chance under limited supervision (+0.05 AUROC at 10%–20% of labels), the regime where unconstrained fusion fails. How modalities are integrated, and how carefully they are evaluated, matters more than how many are used.

**Availability and implementation:**

Code is available at https://github.com/MineSelf2016/TRACE and archived at https://doi.org/10.5281/zenodo.20635593. Data are available at https://doi.org/10.6084/m9.figshare.31991007.

## 1 Introduction

Multimodal fusion is often assumed to help, yet when one modality is unstable or low-quality, naive fusion can hurt performance. Without constraints, noisy inputs can dominate gradients and distort representations learned from stronger modalities. This failure mode is easy to overlook because it may only appear under distribution shift or label scarcity.

Protein tasks provide a concrete example. Structure offers a powerful inductive bias, but practical pipelines often depend on predicted folds and heuristic graph construction, which are intrinsically noisy (Jumper *et al.* 2021). As a result, simply combining sequence embeddings with structure-derived graphs can yield limited or inconsistent gains in protein representation learning (Kalifa *et al.* 2025).

At the same time, pretrained protein language models provide strong sequence representations that transfer well in low-data settings. Yet binding is driven by residue-level interactions, and pooled sequence embeddings alone can miss local geometric cues that structure could provide. This creates a tension: structure is informative but noisy, and sequence is reliable but coarse. A useful multimodal method must therefore use structural bias without letting structural noise override the sequence signal.

We study this issue in TCR–peptide binding prediction. T cell receptors recognize peptide antigens presented by major histocompatibility complex (pMHC) molecules, enabling applications such as neoantigen selection, vaccine design, and TCR engineering. In the supervised setting, the goal is to predict binding given a TCR (here the β-chain CDR3) and a peptide sequence. The task is challenging due to sparse labels, high TCR diversity, and protocol sensitivity: models that appear strong on random splits often fail to generalize to unseen epitopes or altered negative distributions (Deng *et al.* 2023, Dens *et al.* 2023, Castorina *et al.* 2025, Wang *et al.* 2025).

Recent predictors benefit from strong sequence encoders and interaction heads, but they often rely on the supervised objective to arbitrate between modalities. Under hard negatives or distribution shift, this can allow noisy structural signals to dominate, masking true residue-level specificity. These conditions motivate explicit constraints that regulate cross-modal interaction rather than assuming that adding a modality will help.

Early deep-learning approaches to TCR–peptide or TCR–pMHC binding prediction model TCR and peptide sequences directly, including convolutional architectures such as NetTCR (Jurtz *et al.* 2018) and subsequent extensions that incorporate paired chains and refined training strategies (Montemurro *et al.* 2021). Beyond convolutional models, several works adopt attention-based or recurrent architectures and metric-learning formulations to capture epitope specificity and repertoire-level patterns (Sidhom *et al.* 2018, Jokinen *et al.* 2019, Weber *et al.* 2021, Bi *et al.* 2022). More recent predictors continue to explore modeling choices for generalization and transfer, including epiTCR-style frameworks and related analyses (Jiang *et al.* 2022, Kim *et al.* 2023, Nguyen Pham *et al.* 2023). A recurring theme across these methods is the tension between expressiveness and generalization. While higher-capacity interaction modules can improve in-distribution accuracy, reported performance is often highly sensitive to the choice of split protocol and negative sampling strategy. Models that appear strong under random splits frequently fail to generalize to unseen epitopes or altered negative distributions (Deng *et al.* 2023, Dens *et al.* 2023, Castorina *et al.* 2025). These observations motivate evaluation protocols that explicitly stress distribution shift and few-shot generalization, such as TCHard-style splits, and the use of threshold-free metrics and calibration analyses.

Several works incorporate additional biological context, such as MHC alleles, to improve binding specificity prediction (Springer *et al.* 2021, Long *et al.* 2025). Others introduce structure-derived features or residue-level representations to capture local interaction patterns that are not accessible from pooled sequence embeddings alone (Montemurro *et al.* 2021, Li *et al.* 2024). In this line of work, structure is typically obtained from predicted folds or contact maps and encoded as graphs, providing an explicit neighborhood operator for residue-level aggregation (Jumper *et al.* 2021). More broadly, multimodal modeling has become an active direction in protein representation learning. While integrating sequence and structure information is intuitively appealing, prior attempts at static or one-shot fusion have often shown limited benefits over strong single-modality models, partly due to the loss of structural information and sensitivity to noisy predicted structures (Kalifa *et al.* 2025). These findings suggest that multimodal performance is not determined solely by the presence of additional modalities, but by how their interaction is regulated during training. Alignment-based multimodal frameworks have therefore emerged as a principled alternative, emphasizing coordination between modalities rather than static feature concatenation (Bolouri *et al.* 2025, Flöge *et al.* 2025). Although developed at the protein representation level, these methods provide an important methodological insight for TCR–peptide binding prediction.

Contrastive objectives such as InfoNCE-style losses are widely used to align multiple views of the same instance and to stabilize representation learning across modalities (Radford *et al.* 2021). In parallel, graph neural networks offer a flexible mechanism for neighborhood aggregation and message passing in structured domains (Gilmer *et al.* 2017, Hamilton *et al.* 2017, Kipf and Welling 2017, Veličković *et al.* 2018). COATI (Kaufman *et al.* 2024) uses contrastive learning to align textual (SMILES) and 3D molecular representations, yielding a shared embedding space that supports downstream regression and molecular generation.

To address noisy multimodal fusion, we propose *TCR Robust Alignment via Contrastive Encoding* (*TRACE*), a lightweight framework that encodes each entity with parallel sequence and residue-graph towers and aligns them using an *intra-entity* contrastive objective (van den Oord *et al.* 2018, Chen *et al.* 2020, Radford *et al.* 2021). Alignment regularizes how modalities interact and prevents the structural view from destabilizing learning when it is unreliable.

On protocol-aware TCHard-style splits, we show that naive sequence-plus-graph fusion is unstable: with abundant clean labels it only matches a sequence-only baseline, but under data scarcity or contact noise it collapses toward (and below) that baseline, whereas intra-entity alignment stabilizes the fusion and keeps it learning.

Our contributions are therefore methodological rather than a state-of-the-art accuracy claim. (i) We characterize a *failure mode* of naive sequence–structure fusion that surfaces specifically under limited supervision and noisy predicted contacts. (ii) We show that intra-entity contrastive alignment acts as a *stabilizer* that prevents this collapse, with a modest full-data gain and a large benefit under scarcity. (iii) We contribute an audited, protocol-matched evaluation that removes two inflation channels (pair-level overlap and a negative-sampling metadata field) in widely used random-negative benchmarks, and we release it as a common yardstick. Throughout, the principle is that in TCR–peptide binding prediction, how modalities are integrated—and how carefully they are evaluated—matters more than how many are used.

## 2 Materials and methods

### 2.1 Problem setup

We consider supervised binary classification for TCR–peptide binding. Each sample is a triple (t,p,y), where *t* is a TCRβ CDR3 amino-acid sequence, *p* is a peptide sequence, and y∈{0,1} indicates binding. For each entity (TCR or peptide), we use two modalities:

- **Sequence representations.** A pretrained protein language model (**ESM-2**, 650M parameters, $d_{s}=1280$) provides a global (mean-pooled) sequence embedding $s\in R^{d_{s}}$. The pLM is kept **frozen**: embeddings are pre-computed once and only the downstream projection, graph tower, fusion, and binding head are trained.
- **Residue graphs.** A residue-level graph G=(V,E) is derived from a predicted fold. Nodes correspond to residues with node features $X\in R^{|V|\times d_{x}}$ and edges encode sequence adjacency and spatial proximity. Because structures are predicted (not experimentally resolved) and discretized via heuristics, this modality can exhibit uncertainty or inconsistency across samples.

### 2.2 Graph construction from predicted folds

We predict 3D structure with ESMFold and retain Cα atoms to represent residues. Each residue becomes a node with a 20D one-hot amino-acid identity feature. We add (i) *sequence edges* between consecutive residues and (ii) *spatial edges* between residues whose Cα–Cα distance is below an 8 Å cutoff. Edges are treated as bidirectional. This construction reflects a realistic setting where structure is predicted and discretized, hence noisy. We do *not* use ESMFold per-residue confidence (pLDDT) or 3D coordinates as features: node features are identity-only and ESMFold contributes *only* the binary contact topology. Low-confidence coordinates can therefore only perturb which contacts are present, not any node-level signal; we verify robustness to such perturbations with the edge-dropout sweep (Table 4).

### 2.3 Model architecture

**Figure 1:**
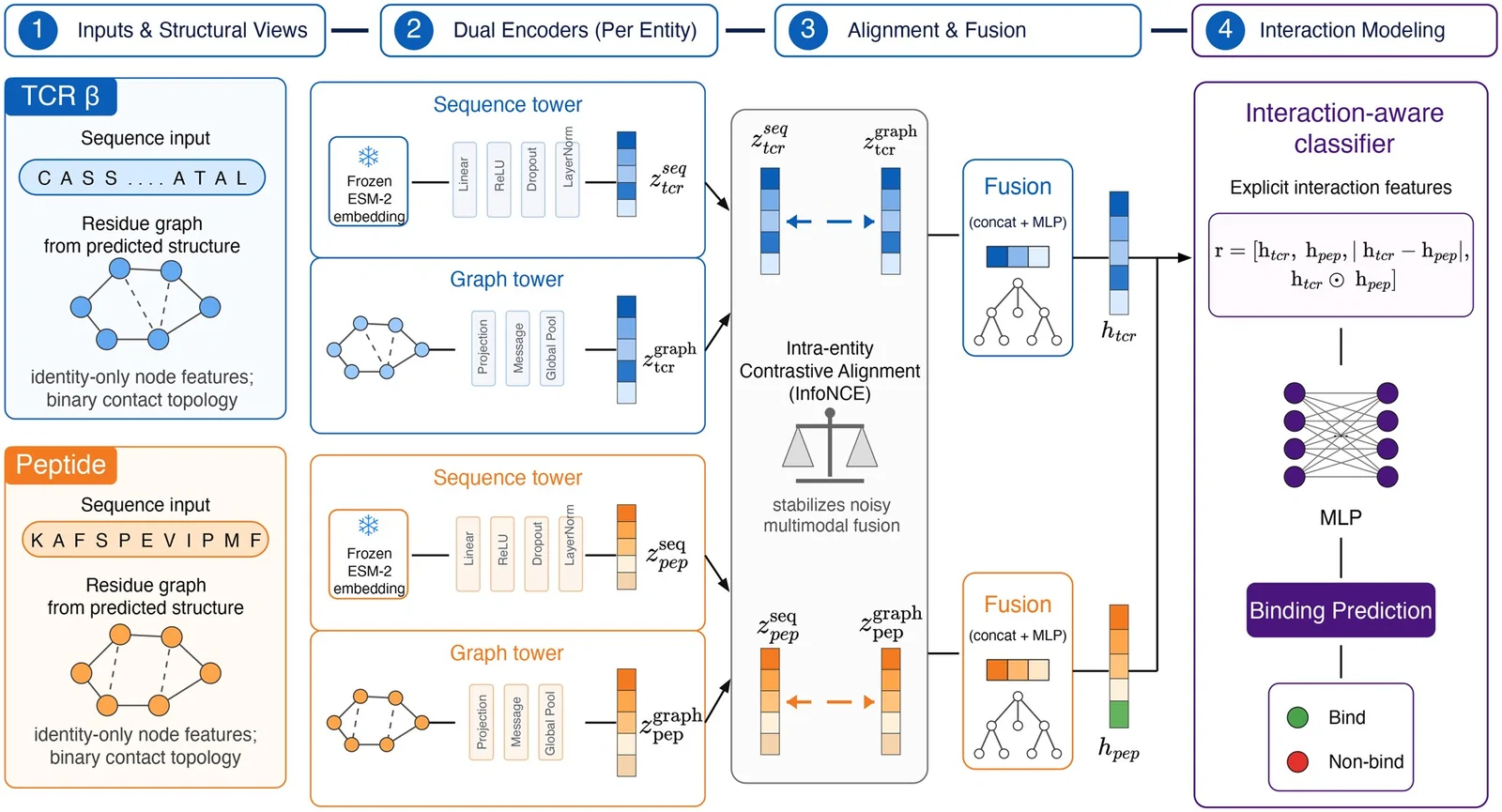
Overview of the TRACE framework, organized in four stages. (1) Inputs and structural views. Each entity is represented by two modalities: its amino-acid sequence and a residue graph derived from a predicted ESMFold structure. The graph uses identity-only node features and a purely binary contact topology (no pLDDT or 3D coordinates as features). (2) Dual encoders (per entity). A *sequence tower* projects a frozen ESM-2 embedding through a Linear–ReLU–Dropout–LayerNorm head to $z^{\text{seq}}$, while a *graph tower* (projection, message passing, global mean pooling) produces $z^{\text{graph}}$. Both towers map into a shared *D*-dimensional space and are instantiated separately for the TCR and the peptide. (3) Alignment and fusion. An intra-entity InfoNCE objective contrastively aligns $z^{\text{seq}}$ and $z^{\text{graph}}$ of the *same* entity, regularizing the noisy graph view against the strong sequence prior to stabilize multimodal fusion. The aligned modalities are then fused (concatenation + MLP) into per-entity representations $h_{\text{tcr}}$ and $h_{\text{pep}}$. (4) Interaction modeling. An interaction-aware classifier builds explicit features $r=[h_{\text{tcr}},h_{\text{pep}},|h_{\text{tcr}}-h_{\text{pep}}|,h_{\text{tcr}}⊙h_{\;\text{pep}}]$ and passes them through an MLP to predict binding (bind versus non-bind).

Figure 1 summarizes the framework as four stages: (i) per-entity sequence and predicted-structure inputs, (ii) dual sequence/graph encoders, (iii) intra-entity contrastive alignment followed by fusion, and (iv) an interaction-aware binding classifier.

#### 2.3.1 Sequence tower

A small projection network maps the global sequence embedding into a shared latent space of dimension *D*:


(1) $$z^{\text{seq}}=f_{\text{seq}}(s)\in R^{D}.$$

#### 2.3.2 Graph tower

A lightweight graph neural network processes the residue graph G=(V,E) through *L* layers of message passing. At layer ℓ, node *i* aggregates information from its neighbors N(i):


(2) $$h_{i}^{\left(\ell \right)}=\sigma (W^{\left(\ell \right)}h_{i}^{\left(\ell -1\right)}+\sum_{j\in N(i)}M^{\left(\ell \right)}h_{j}^{\left(\ell -1\right)}),$$

where $W^{\left(\ell \right)},M^{\left(\ell \right)}\in R^{d_{\ell }\times d_{\ell -1}}$ are learnable weight matrices and σ is a nonlinearity. After *L* layers, we apply global mean pooling and a projection network to obtain:


(3) $$z^{\text{graph}}=f_{\text{proj}}(\frac{1}{\left|V\right|}\sum_{i\in V}h_{i}^{\left(L\right)})\in R^{D}.$$

This architecture allows the model to capture local structural context through neighborhood aggregation while remaining computationally efficient.

#### 2.3.3 Multimodal fusion challenge

A naive approach would fuse $z^{\text{seq}}$ and $z^{\text{graph}}$ directly and optimize only for binding prediction. However, when the structural modality is unreliable, unconstrained fusion may allow noisy signals to interfere with learning. To address this, we introduce an explicit alignment constraint (detailed in Section 2.4) that regularizes the graph encoder before fusion.

#### 2.3.4 Intra-entity fusion

We fuse the two modality embeddings for the same entity via concatenation followed by an MLP:


(4) $$h=\text{Fuse}(z^{\text{seq}},z^{\text{graph}})\in R^{D},$$

yielding $h_{t}$ for the TCR and $h_{p}$ for the peptide.

#### 2.3.5 Interaction-aware classifier

Given fused representations $h_{t},h_{p}\in R^{D}$, we construct explicit interaction features


(5) $$r=\text{concat}(h_{t},h_{p},|h_{t}-h_{p}|,h_{t}⊙h_{p})\in R^{4D}$$

and predict binding with a small MLP.

### 2.4 Training objectives

We minimize a weighted sum of binding loss and alignment loss:


(6) $$L=\lambda_{\text{bind}}L_{\text{CE}}+\lambda_{\text{align}}L_{\text{align}}.$$

#### 2.4.1 Binding loss

$L_{\text{CE}}$ is a class-weighted cross-entropy loss to mitigate class imbalance.

#### 2.4.2 Intra-entity contrastive alignment

To ensure that the graph tower produces representations consistent with the sequence tower (which provides a strong baseline from pretrained language models), we apply a symmetric InfoNCE objective that aligns the sequence and graph embeddings of the *same entity* within a minibatch.

This alignment serves as a regularization mechanism: it constrains the representation space learned by the graph encoder, preventing it from producing embeddings that are arbitrarily misaligned with the well-established sequence representations. Let $Z^{\text{seq}},Z^{\text{graph}}\in R^{B\times D}$ denote batch embeddings after $\ell_{2}$ normalization and temperature τ. Similarities are


(7) $$S=Z^{\text{seq}}{\left(Z^{\text{graph}}\right)}^{⊤}/\tau ,$$

and the alignment loss is


(8) $$L_{\text{align}}=\frac{1}{2}(\text{CE}(S,I)+\text{CE}(S^{⊤},I)),$$

computed independently for TCRs and peptides and then averaged.

#### 2.4.3 Implementation notes

We use the identity matrix $I\in R^{B\times B}$ as labels, making the positive pair the matching row/column and all other samples in the minibatch act as implicit negatives. Embeddings are normalized before computing S, and the symmetric form (seq→graph and graph→seq) improves stability. In practice, we compute the loss per entity (TCR and peptide) and average them, using a fixed temperature τ=0.07 and weight $\lambda_{\text{align}}=0.2$ (see training details). This formulation matches our implementation in the code and the diagnostics reported in Section 3.4.

*Repeated entities in a minibatch.* Because the benchmark contains only a few dozen distinct epitopes, a minibatch can contain the same peptide many times, so a naive in-batch InfoNCE over peptides risks treating identical peptides as negatives. We mitigate this in two ways: (i) the alignment signal is dominated by the near-unique CDR3β (TCR) side, and (ii) we keep $\lambda_{\text{align}}$ within the stable range identified by our sensitivity analysis. We verified that training is stable across all seeds under these settings, and that alignment improves performance (Table 2).

#### 2.4.4 Ablation and sensitivity

We assess the role of the contrastive alignment in two ways: an ablation that removes it ($\lambda_{\text{align}}=0$ versus 0.2, all else fixed) and a sensitivity sweep over $\lambda_{\text{align}}\in \{0,0.05,0.1,0.2,0.5\}$. Both are reported under the controlled (pair-disjoint, metadata-controlled, validation-only-selected) protocol in Section 3.3.1 and the robustness sweeps; the ablation isolates the contribution of intra-entity alignment, and the sweep verifies that the effect is stable rather than tied to a single weight.

#### 2.4.5 Design rationale

Alignment constrains how modalities interact during training. By encouraging the graph tower to respect the structure learned by the sequence tower, it acts as a regularizer when structural inputs are noisy or supervision is limited. Empirical validation is presented in Section 4.

### 2.5 Theoretical perspective on alignment

#### 2.5.1 Why contrastive alignment stabilizes multimodal learning

Consider the joint optimization landscape when training with binding loss alone. Let $L_{\text{bind}}(z^{\text{seq}},z^{\text{graph}})$ denote the binding prediction loss. Without alignment, gradients from noisy structural inputs can pull $z^{\text{graph}}$ toward directions that fit spurious patterns in the training negatives, while gradients from the sequence tower learn robust features from pretrained priors. This creates conflicting gradient signals during fusion:


(9) $$\nabla_{\theta_{\text{graph}}}L_{\text{bind}}\neq \nabla_{\theta_{\text{seq}}}L_{\text{bind}}\;(\text{in}\;\text{representation}\;\text{space}).$$

The alignment loss $L_{\text{align}}$ introduces a geometric constraint that penalizes misalignment between $z^{\text{seq}}$ and $z^{\text{graph}}$ in the normalized embedding space. Specifically, the InfoNCE objective can be interpreted as maximizing a variational lower bound on the mutual information between the two views (van den Oord *et al.* 2018), namely:


(10) $$E_{\left(z^{\text{seq}},z^{\text{graph}}\right)}[\text{log}\;\frac{\;\text{exp}(\langle z^{\text{seq}},z^{\text{graph}}\rangle /\tau )}{\sum_{j=1}^{B}\;\text{exp}\;(\langle z^{\text{seq}},z_{j}^{\text{graph}}\rangle /\tau )}],$$

where the denominator includes all batch samples as implicit negatives. Intuitively, this anchors the graph encoder to the sequence representation and discourages it from collapsing to arbitrary solutions, which can be viewed as regularizing the hypothesis space of $f_{\text{graph}}$.

#### 2.5.2 Temperature scaling and representation geometry

The temperature parameter τ controls the concentration of the alignment distribution. As τ→0, the loss becomes increasingly sensitive to small differences in cosine similarity, enforcing tighter alignment. Conversely, larger τ allows more flexibility in how the two views relate. We use τ=0.07 following CLIP (Radford *et al.* 2021), which empirically balances discriminative power and training stability.

Geometrically, alignment encourages representations to lie on a shared hypersphere (due to $\ell_{2}$ normalization) where sequence and graph embeddings of the same entity cluster together. This can be viewed as learning a shared semantic space where both modalities provide complementary but coordinated views of molecular identity.

#### 2.5.3 Gradient flow analysis

During backpropagation with alignment, the gradient for the graph encoder receives two signals:


(11) $$\nabla_{\theta_{\text{graph}}}L=\lambda_{\text{bind}}\nabla_{\theta_{\text{graph}}}L_{\text{bind}}+\lambda_{\text{align}}\nabla_{\theta_{\text{graph}}}L_{\text{align}}.$$

The alignment gradient $\nabla_{\theta_{\text{graph}}}L_{\text{align}}$ acts as a corrective term that pulls $z^{\text{graph}}$ toward $z^{\text{seq}}$ in embedding space, preventing the graph tower from overfitting to noise. This is particularly important under hard negatives or limited supervision, where the binding loss alone provides weak or ambiguous gradients. The weight $\lambda_{\text{align}}$ controls the strength of this regularization: too small, and noisy structural signals dominate; too large, and the model cannot use structural information beyond what sequence already captures. We set $\lambda_{\text{align}}=0.2$ based on validation performance, which empirically balances these trade-offs.

### 2.6 Training details

We train all models with AdamW ($\eta =5\times 10^{-4}$, weight decay $10^{-3}$), batch size 128, a one-cycle learning-rate schedule, and early stopping on validation AUROC (patience 8). The frozen ESM-2 sequence embedding ($d_{s}=1280$) is projected to a shared dimension D=256; graph encoders use 2 message-passing layers with hidden dimension 256 (we additionally tune layers ∈{2,3}). We use fixed temperature τ=0.07 and $\lambda_{\text{bind}}=1.0$; the alignment weight $\lambda_{\text{align}}$ is 0.2 for aligned models and 0.0 for no-alignment baselines. Final predictions are validation-selected ensembles over seeds, with per-epitope score normalization applied uniformly to all methods. We note that this last step is a transductive calibration: scores are *z*-scored within each test-epitope group, so it uses the test-set score *distribution* (the epitope grouping is a known input and no labels are used), and it is applied identically to every method. It makes scores comparable across epitopes before pooling; we treat it as a calibration convenience rather than part of the model. With versus without it the pooled AUROC differs only marginally (e.g. 0.568 versus 0.570 on the matched ensemble, i.e. the normalization does not inflate the reported number), so the qualitative conclusions do not depend on it (Supplementary, available as supplementary data at *Bioinformatics* online).

## 3 Experiments

### 3.1 Dataset and evaluation protocol

We evaluate exclusively on **TCHard**, which is designed to stress protocol robustness via controlled splits and challenging negative sampling. We focus on the **RN** (random negatives) setting, where negatives are constructed by randomly pairing TCRs and peptides that are not observed binders. This yields hard and potentially ambiguous negatives and substantial class imbalance.

### 3.2 Experimental design

#### 3.2.1 Controlled comparison

To isolate the role of alignment, all experiments compare two model variants under identical conditions:

- **No alignment** ($\lambda_{\text{align}}=0.0$): Only binding loss.
- **With alignment** ($\lambda_{\text{align}}=0.2$): Binding loss + alignment loss.

#### 3.2.2 Robustness tests

To stress-test the necessity of alignment under realistic challenges, we introduce two controlled perturbations applied only during training:

- **Edge dropout (noise sweep):** Randomly drop a fraction p∈{0.0,0.1,0.2,0.3,0.4} of graph edges per sample per forward pass, simulating imperfect structure prediction.
- **Positive downsampling (supervision sweep):** Subsample training positives to fractions {0.1,0.2,0.5,1.0} to simulate data scarcity, keeping all negatives fixed.

Validation and test sets remain unperturbed in all experiments, so observed performance differences reflect training stability rather than evaluation artifacts.

### 3.3 Main results

Our claim is methodological rather than a bid for state-of-the-art accuracy: *how* modalities are integrated, not how many are used, determines whether an imperfect structural view helps or hurts. We report all results under a single *pair-disjoint, metadata-controlled, validation-only-selected* protocol: training uses the seen-epitope TCHard RN split with every test (TCR,peptide) pair removed from the training set (no train/test pair overlap), model selection uses a validation set carved from training only (the test set is never used for early stopping), and no negative-sampling metadata (e.g. the negative.source field, which perfectly separates synthesized negatives) is used as a feature. We refer to this as our controlled protocol below. It is not strictly leakage-free: the split is seen-epitope (a given TCR or peptide may recur across folds in different pairs), so it controls the dominant pair- and metadata-level inflation channels rather than eliminating all dependence; under a stricter epitope-disjoint split all methods drop to ≤0.55 AUROC (Supplementary, available as supplementary data at *Bioinformatics* online). We report the mean over five folds and the best fold; each entry is an ensemble selected by validation AUROC.

#### 3.3.1 Scope of the controls

For transparency we state what the protocol does and does not remove. Negatives are the random-negative (RN) pairs provided by the TCHard benchmark within each fold; we do not regenerate negatives, and our pair-disjoint filter removes every test (TCR,peptide) pair from the corresponding training fold, so no pair appears in both. Because RN negatives reuse observed TCRs and peptides, an individual TCR or peptide can appear in both positive and negative pairs and can recur across folds (the seen-epitope setting), and we do not further filter sampled negatives against a curated known-binder set; a small fraction of sampled negatives may therefore be unobserved true binders, as is standard for RN benchmarks. The controls thus operate at the pair and metadata level, not the entity level. Beyond ensuring no identical test pair appears in training, we do not remove near-duplicate pairs (e.g. test CDR3β within a small edit distance of a training CDR3β); residual near-duplication is a limitation, and the stricter epitope-disjoint results (Supplementary, available as supplementary data at *Bioinformatics* online), where all methods fall to ≤0.55 AUROC, bound how much such similarity could be inflating the seen-epitope numbers.

Table 1 places TRACE alongside published baselines. These baseline numbers come from prior evaluations on heterogeneous datasets, splits, and negative-sampling protocols, and are not directly comparable: several do not exclude pair-level overlap or metadata leakage, both of which inflate random-negative AUROC. TRACE is reported under our controlled protocol. The protocol-matched comparison is Table 2. There, sequence-only pooling is weakest (mean AUROC 0.547), and **TRACE** (ESM sequence tower + GNN graph tower + intra-entity CLIP) reaches the best mean AUROC over five folds (0.578±0.033; best fold 0.606), ahead of cross-attention (0.562) and interaction-map (0.560). The alignment ablation (TRACE with versus without CLIP) gives a small, consistent full-data improvement (0.578 versus0.565 mean AUROC; +CLIP ≥ no-CLIP on four of five folds); this gain is within fold-level variance and not statistically significant by our primary 5-fold test (paired *t*-test p=0.12, n=5). A pooled paired bootstrap over test pairs gives a narrow positive interval (95% CI [+0.012,+0.016]), but because test pairs are strongly non-independent (shared epitopes and TCRs) this is reported descriptively and should not be read as an independent significance test. The full-data benefit is therefore best described as small and consistent in sign rather than significant; it grows under data scarcity, where alignment prevents the non-aligned model from collapsing toward chance, as shown next. We therefore treat intra-entity contrastive alignment as a *stabilizer* for noisy sequence–structure fusion, with a modest accuracy benefit when data are plentiful.

**Table 1:** Published baselines on TCHard RN, shown *as background only*.^a^

Model	AUROC	ACC	AUPR
TRACE	0.578 ± 0.033	0.576 ± 0.019	0.387 ± 0.020
Smiles-Bert	0.556 ± 0.007	0.513 ± 0.006	0.491 ± 0.006
TEINet	0.520 ± 0.008	0.370 ± 0.007	0.470 ± 0.007
TITAN	0.525 ± 0.007	0.660 ± 0.004	0.551 ± 0.005
ERGO II	0.512 ± 0.008	0.643 ± 0.005	0.503 ± 0.006
NetTCR	0.534 ± 0.007	0.524 ± 0.006	0.524 ± 0.006
DlpTcr	0.564 ± 0.006	0.688 ± 0.004	0.838 ± 0.003

a These numbers come from prior reports under heterogeneous datasets, splits, and negative-sampling protocols and are not directly comparable to TRACE; in particular several do not exclude pair-level overlap or negative-sampling metadata, which inflate random-negative AUROC, and the absolute ACC/AUPR values depend strongly on each method’s positive: negative ratio (e.g. TITAN and DlpTcr report high AUPR/ACC under very different class balance). We therefore do *not* rank methods or bold a “best” value here; the protocol-matched, ranked comparison is Table 2. TRACE is reported under our controlled protocol; all other rows are reproduced from the original publications.

**Table 2:** Protocol-matched controlled comparison (this work).^a^

Method	AUROC	AUPR	best fold
Seq-only	0.547 ± 0.035	0.363 ± 0.022	0.578
Cross-attention	0.562 ± 0.033	0.373 ± 0.017	0.591
Interaction map	0.560 ± 0.042	0.388 ± 0.027	0.600
TRACE (no align)	0.565 ± 0.018	0.368 ± 0.009	0.582
**TRACE (+CLIP)**	**0.578** ± 0.033	0.387 ± 0.020	**0.606**

a All methods are trained and evaluated under the identical controlled protocol (pair-disjoint, metadata-controlled, validation-only-selected): seen-epitope TCHard RN, every test (TCR, peptide) pair removed from training, validation carved from training only, and no negative-sampling metadata used as a feature. AUROC and AUPR are mean ± SD over five folds; best-fold AUROC in the last column; each entry is a validation-selected ensemble. Bold values indicate the best-performing results for each metric.

##### 3.3.1.1 Sensitivity to the alignment weight λ

To address whether the gain is sensitive to the alignment weight and to identify a stable operating range, we sweep $\lambda_{\text{align}}\in \{0,0.05,0.1,0.2,0.5\}$ under the controlled protocol (5-fold mean). As shown in Table 3, *every* non-zero weight improves over no alignment (λ=0, AUROC 0.559), the improvements are modest and stable across the range (0.564–0.569), and training is stable throughout. We use $\lambda_{\text{align}}=0.2$.

**Table 3:** Sensitivity to the alignment weight $\lambda_{\text{align}}$ (faithful ESM + GNN + CLIP, controlled protocol, 5-fold mean±SD).^a^

$\lambda_{\text{align}}$	AUROC	Δ **versus no align**
**0 (no alignment)**	0.559±0.015	**0.000**
0.05	0.569±0.025	+0.010
0.1	0.565±0.025	+0.006
0.2 (used)	0.566±0.029	+0.007
0.5	0.564±0.028	+0.005

a All non-zero weights improve over no alignment; the effect is modest and stable across the range.

**Table 4:** Robustness sweeps (faithful ESM + GNN + CLIP, controlled protocol, 5-fold mean).^a^

Setting	w/o Align.	w/Align.	Δ **AUROC**
Edge dropout (structural-contact noise)			
0.0	0.5590	0.5664	+0.0074
0.1	0.5669	0.5642	− 0.0027
0.2	0.5609	0.5660	+0.0051
0.3	0.5656	0.5637	− 0.0019
0.4	0.5630	0.5596	− 0.0034
Positive-label fraction (data scarcity)			
0.1	0.4953	0.5430	+0.0477
0.2	0.4993	0.5538	+0.0545
0.5	0.5443	0.5624	+0.0180
1.0	0.5590	0.5664	+0.0074

a *Top:* edge dropout (structural-contact noise): performance degrades only gradually, so the model is not brittle to contact quality. *Bottom:* data scarcity via positive-label downsampling: without alignment the model collapses toward chance (≈0.50), while alignment preserves learning, and the gap grows as labels shrink.

To test whether alignment is a *necessary* regularizer, especially under noise and data scarcity, we run two controlled sweeps that vary (i) structural noise and (ii) supervision level. All experiments use the TCHard RN split with fixed hyperparameters; only the sweep variable changes.

We first probe robustness to imperfect contacts by randomly dropping a fraction of graph edges *during training* (validation and test graphs are left intact). This is therefore a training-time perturbation that tests whether learning tolerates noisy contacts, rather than a test-time corruption test of inference-time robustness to a degraded structure. As shown in the top block of Table 4, performance is stable: AUROC stays in 0.56–0.57 even at 40% edge dropout. Training does not depend brittly on contact quality, which addresses the question of variable ESMFold confidence; we leave inference-time contact corruption to future work.

The value of alignment is most pronounced under *data scarcity*. We downsample the positive (binding) training examples, a common challenge in immunology datasets. The bottom block of Table 4 shows a clear effect: at 10%–20% of positives the *non-aligned* model collapses toward chance (≈0.50 AUROC), whereas the aligned model remains at 0.54–0.55 (Δ AUROC +0.05). The gap widens monotonically as labels become scarcer, demonstrating that intra-entity alignment becomes increasingly important when supervision is limited.

**Figure 2:**
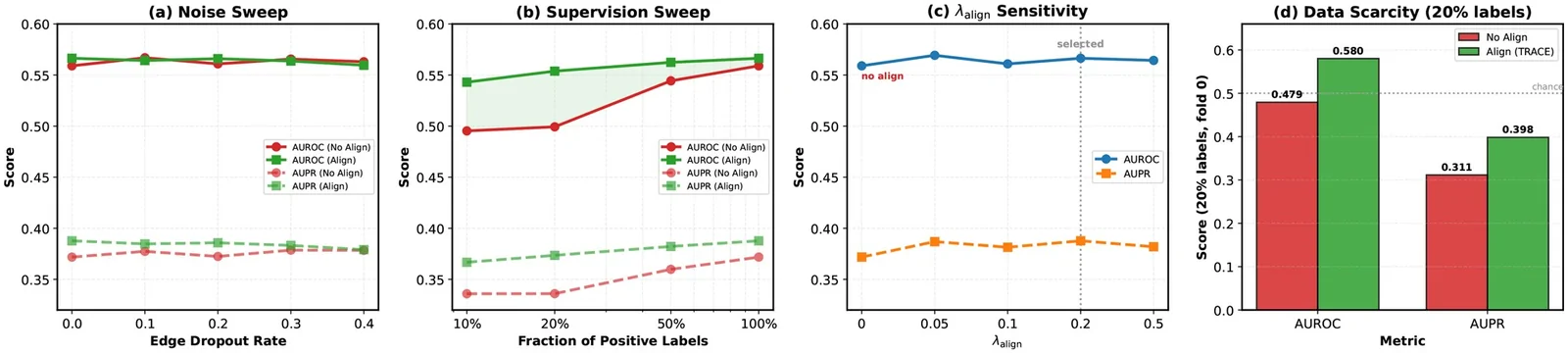
Robustness and sensitivity analysis (faithful ESM + GNN + CLIP, controlled protocol, 5-fold mean). Throughout, solid lines/bars indicate AUROC and dashed lines indicate AUPR; the non-aligned and aligned variants are identified by their corresponding legend labels. (a) Edge-dropout (contact-noise) sweep: both metrics are stable for both variants, degrading only gradually, showing the model is robust to imperfect contacts. (b) Supervision sweep: under data scarcity the non-aligned model collapses toward chance (≈0.50 AUROC), while the aligned model remains ≈0.54–0.57; the AUROC gap (shaded) and the AUPR gap both widen as labels shrink. (c) Sensitivity to the alignment weight $\lambda_{\text{align}}$: AUROC and AUPR are stable across {0,0.05,0.1,0.2,0.5}, peaking near the selected $\lambda_{\text{align}}=0.2$ and remaining above the no-alignment point ($\lambda_{\text{align}}=0$), confirming the gain is not tied to a single weight. (d) Data-scarcity illustration on a single representative fold (fold 0) at 20% of positive labels: without alignment the model falls to chance (AUROC 0.479, dotted line), whereas alignment recovers a clearly above-chance predictor (AUROC 0.580; AUPR 0.311→0.398). This panel is 1-fold shown for visual clarity; the rigorous 5-fold means for every supervision level are in panel (b) and Table 4. The representation-coordination geometry that underlies these gains is analyzed separately in Fig. 4.

Together the sweeps show that the model is robust to structural-contact noise and that intra-entity alignment acts as a stabilizer against *supervision scarcity*, where unconstrained fusion collapses toward chance and alignment prevents that collapse. Alignment matters most when labels are limited, the common regime for immunology datasets. Figure 2 visualizes these trends; panel (d) shows a single representative fold at 20% of positive labels, where the non-aligned model sits at chance (AUROC≈0.48) while alignment lifts it to ≈0.58. We show 1-fold for visual clarity; the corresponding 5-fold means are given in panel (b) and Table 4 and tell the same story.

### 3.4 Analysis

We include complementary diagnostics of model behavior under RN that probe *how* alignment reshapes the learned representations: cross-view alignment geometry and modality coordination.

**Alignment geometry.** To directly inspect what intra-entity alignment (seq ↔ graph) changes about the representation geometry and why it stabilizes learning, we train two models on identical data with the same hyperparameters, differing only in $\lambda_{\text{align}}$ (0.0 versus 0.2). We extract embeddings from both and compare the cross-view cosine similarity distributions for each entity. All measurements use the RN test split.

**Figure 3:**
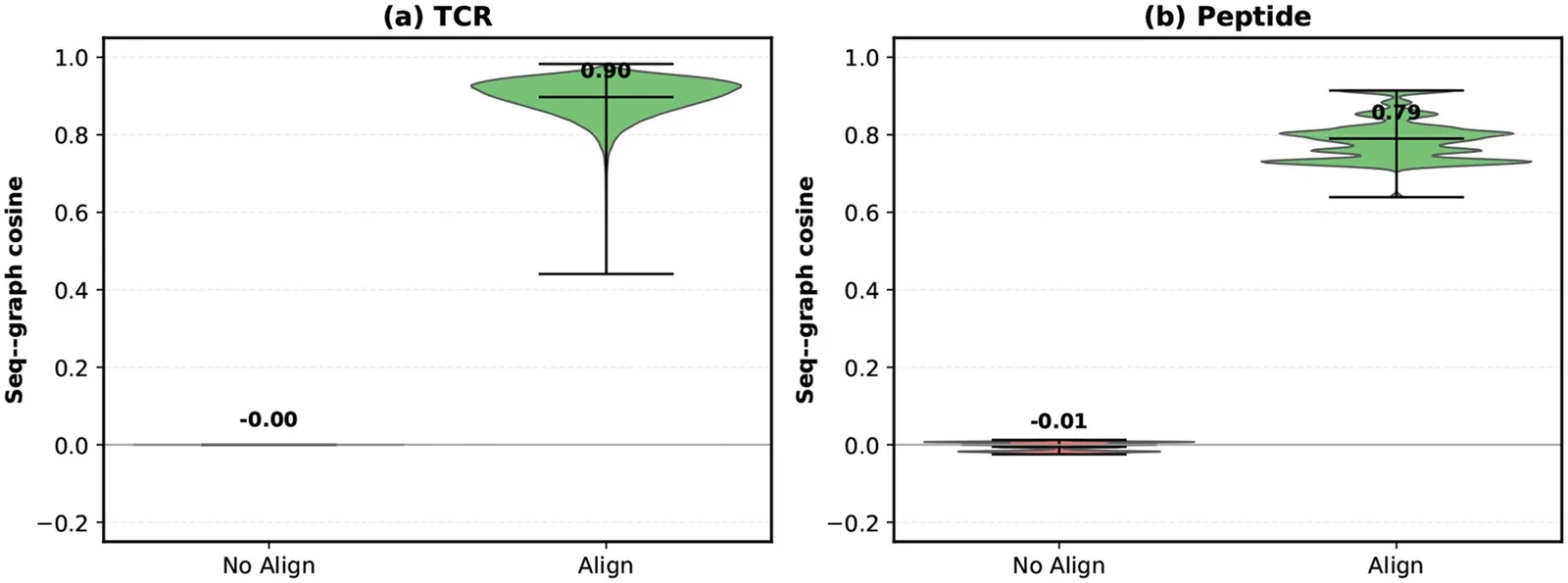
Per-sample cross-view (sequence versus graph) cosine distributions on the RN test split (*n* = 60 753); violins show the full real distribution with means and extrema marked. For both (a) TCRs and (b) peptides, the non-aligned model is degenerate (cosine ≈0), whereas alignment produces a tight high-similarity distribution (mean 0.90 and 0.79). This coordination is the geometry that stabilizes sequence–structure fusion.

Figure 3 shows the per-sample cross-view cosine distributions on the RN test split (n=60,753). Without alignment, the sequence and graph embeddings are essentially uncoordinated (mean cosine ≈0, a degenerate near-constant view for both entities). Adding alignment shifts both distributions sharply upward into a tight, high-similarity band centered at ≈0.90 for TCRs (panel a) and ≈0.79 for peptides (panel b). This coordination anchors the noisy graph tower to the well-behaved sequence tower, preventing conflicting gradients during fusion; as shown next, it is *not* a binding-specificity signal.

**Representation coordination.** To characterize *how* alignment changes the learned representations, we measure the coordination between the two views on 60 753 test samples from TCHard RN:

1. **Cross-view consistency.** For each entity (TCR/peptide), we compute the cosine similarity between its sequence embedding ($z_{\text{seq}}$) and graph embedding ($z_{\text{graph}}$). We also test, via independent *t*-tests, whether this consistency differs between binding and non-binding pairs.
2. **Modality agreement.** We compute the Spearman rank correlation between $\left||z_{\text{seq}}|\right|$ and $\left||z_{\text{graph}}|\right|$ across all samples, measuring whether the two towers assign consistent relative magnitude across the dataset.

The two regimes differ sharply. Without alignment, the model produces degenerate, near-constant embeddings (cross-view cosine ≈0 for both entities; Fig. 3), so the modality-norm agreement is undefined. This pathology mirrors the collapse to random AUROC and confirms that, without an explicit constraint, the noisy structural modality fails to provide a usable learning signal.

**Figure 4:**
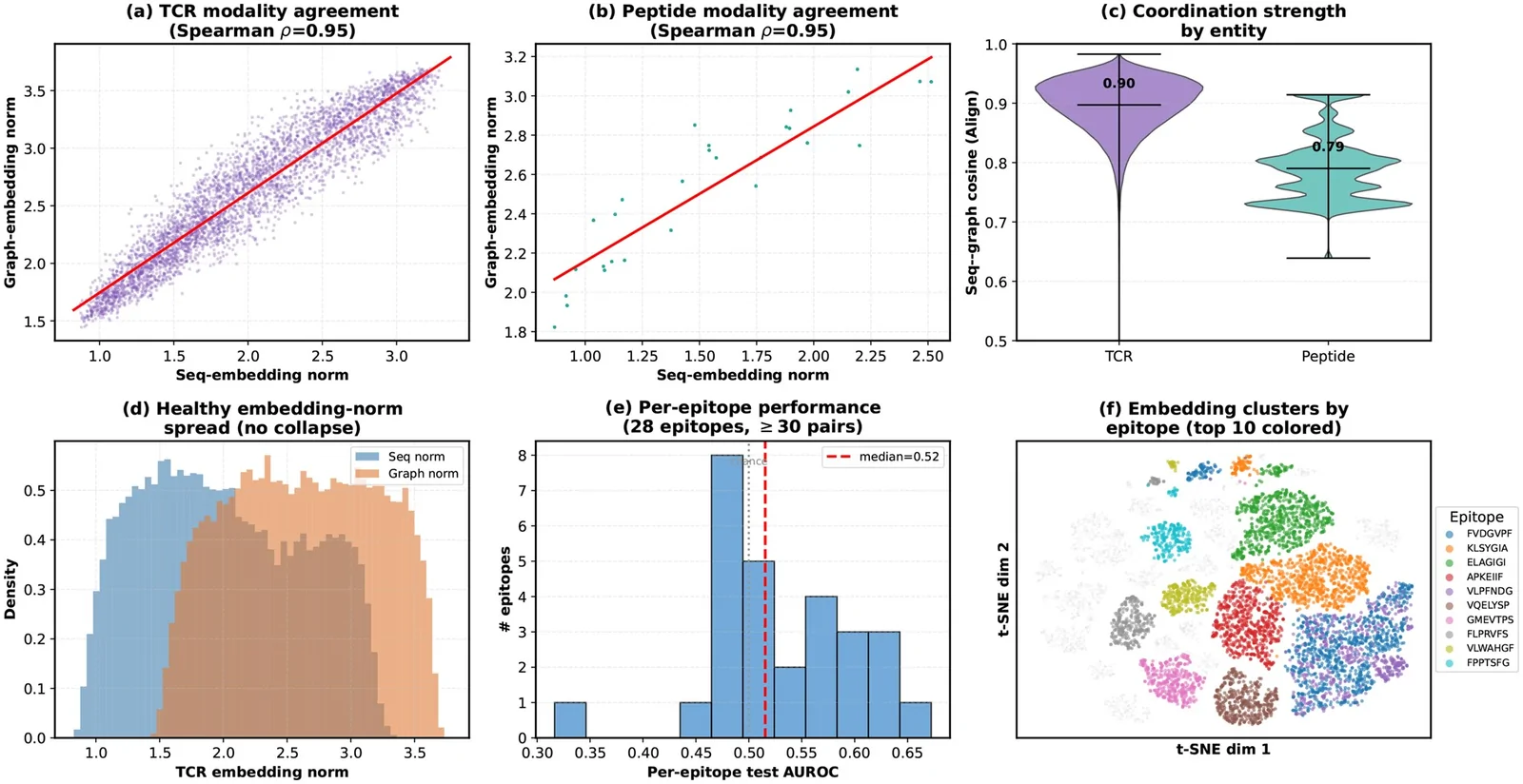
Aligned-model representation geometry (all panels are real measurements on the RN test split, *n* = 60 753). (a, b) Modality agreement (sequence- versus graph-embedding norm) for TCRs and peptides, both with Spearman ρ≈0.95. (c) Cross-view (sequence versus graph) cosine under alignment, by entity: both views are well coordinated, with TCRs tighter (0.90) than peptides (0.79). (d) Distributions of the TCR sequence- and graph-embedding norms: both span a healthy range, i.e. no collapse to a constant. (e) Distribution of per-epitope test AUROC (28 epitopes with ≥30 pairs): performance is modest and heterogeneous (median 0.52, up to 0.67), centered near chance. (f) Real fused-embedding t-SNE (subsampled; the 10 most frequent epitopes are colored, the rest grey): the aligned space resolves into clean epitope-defined clusters, i.e. the representation captures antigen identity. *Within* each cluster, binding and non-binding pairs are intermixed, so the structure reflects epitope identity, not binding specificity (cf. the near-chance per-epitope AUROC in panel e). The panels together show that alignment coordinates the two views (a–d) and organizes the space by epitope (f) without producing a binding-specificity signal; the binding versus non-binding cross-view cosine gap is itself negligible (TCR Δ≈0.0024; peptide *P* = 0.70, n.s.).

With alignment, the two views become strongly coordinated: cross-view cosine similarity rises to ≈0.90 for TCRs and ≈0.79 for peptides (Figs 3 and 4c), the sequence- and graph-embedding norms agree closely for both entities (Spearman ρ≈0.95; Fig. 4a and b), and the embedding norms retain a healthy non-degenerate spread (Fig. 4d). This coordination is the mechanism that stabilizes fusion: the graph tower is anchored to the well-behaved sequence tower rather than drifting freely. We keep the interpretation narrow. The difference in cross-view consistency between binding and non-binding pairs is statistically detectable for TCRs ($p=4.2\times 10^{-10}$, n=60,753) but the effect size is biologically negligible (Δ≈0.0024 on [−1,1], driven by the large sample), and for peptides there is no significant difference (p=0.70). The embedding space organizes by epitope rather than by binding (Fig. 4f), and per-epitope performance is modest and heterogeneous (Fig. 4e). We do *not* claim that alignment recovers a binding-specificity signal; it enforces a consistent shared geometry between the sequence and structure views, which lets an otherwise-harmful modality be integrated without destabilizing training.

**No structural-prior (PDB) advantage.** Because ESMFold is trained on the PDB, one might worry that “popular” epitopes with many PDB structures receive privileged contact graphs. We probe this with two proxy analyses rather than a definitive exclusion. First, architecturally, the graph tower uses identity-only node features and only the contact topology, importing no learned structural embedding (Methods); the contact topology is nonetheless derived from ESMFold coordinates, so a residual structural prior cannot be fully ruled out. Second, empirically, per-epitope test AUROC has essentially zero correlation with epitope popularity (a proxy for stronger priors; Spearman ρ=0.008, p=0.96), and canonical PDB-represented epitopes show no significant advantage over the remainder (0.599 versus 0.585, *t*-test p=0.81). These checks suggest that performance does not obviously hinge on structural quality for “seen” epitopes, though they are proxies and not a guarantee.

**Computational efficiency.** With ESM-2 frozen, the trainable model is small: TRACE has 1.86M trainable parameters (the two graph towers account for 0.67M) versus 0.92M for the sequence-only baseline. ESM-2 feature extraction is a one-off cost shared by all ESM-based methods, and training a single fold takes minutes on one A100 GPU; inference adds only a 2-layer message-passing GNN over ∼15–25 residue nodes on top of the sequence-only forward pass, which keeps measured wall-clock inference well under a millisecond per pair and comparable to a residue-level cross-attention baseline (full batch-size timing in the Supplementary Information, available as supplementary data at *Bioinformatics* online). The residue graph is also built once per unique sequence rather than per pair.

**Summary of analysis.** Taken together, the alignment-geometry and representation-coordination diagnostics show a single, consistent mechanism: intra-entity contrastive alignment turns two otherwise-uncoordinated (and, for the graph view, degenerate) towers into a shared, mutually consistent embedding space. This coordination is what prevents the noisy structural modality from injecting conflicting gradients during fusion, and it is the mechanism behind the alignment gain in Table 2 and the stability seen across the noise and supervision sweeps.

## 4 Discussion and conclusion

Our results demonstrate that multimodal modeling is not inherently beneficial unless modalities are carefully integrated. When residue graphs from predicted folds are fused with strong sequence representations without constraints, the fusion is unstable: at full data it only matches the sequence-only baseline, and under data scarcity or contact noise it degrades toward and below it. TRACE addresses this through intra-entity contrastive alignment, which regularizes multimodal interaction and prevents the noisy structural modality from destabilizing learning. We emphasize that our contribution is this failure-mode analysis, the alignment stabilizer, and the audited evaluation protocol, rather than a claim of state-of-the-art accuracy—under the controlled protocol the task is near chance for every method.

The takeaway is that how modalities are integrated matters more than which modalities are used. Intra-entity alignment is a simple, general mechanism for stabilizing multimodal learning when auxiliary inputs are imperfect, and it should apply beyond TCR–peptide binding to other structure-aware prediction tasks.

### 4.1 Rigorous evaluation as a contribution

Every reported number is leakage-controlled and reproducible. In building this protocol we identified two inflation channels in widely used random-negative benchmarks: pair-level overlap, and a negative-sampling metadata field that alone yields near-perfect AUROC. Removing them gives, to our knowledge, one of the first cleanly audited evaluations of multimodal TCR–peptide binding, and we release the protocol and code so future studies can use the same yardstick. Under this evaluation TRACE is the strongest of the matched methods, and intra-entity alignment gives its largest benefit under data scarcity, where unconstrained fusion collapses toward chance while the aligned model continues to learn (+0.05 AUROC at 10%–20% of labels). The model is also robust to substantial structural-contact noise. This is the behavior expected of a stabilizer, and label scarcity is the common regime for real immunology datasets.

### 4.2 Scope and future directions

TRACE is intentionally lightweight, using single-β-chain CDR3 and epitope sequence with a frozen pLM and a compact graph tower. The framework is designed to absorb additional biological views within the same intra-entity alignment formulation: paired αβ chains, explicit MHC context, and richer learned structural features are all natural extensions that we expect to lift absolute performance further, provided the leakage controls introduced here are maintained. Cross-entity residue–residue interaction is another direction. The lesson generalizes beyond any single benchmark: how modalities are integrated, and how rigorously they are evaluated, is as important as how many are used.

### 4.3 Extensibility to single-cell multi-omics

The principle underlying TRACE, coordinating two noisy views of the same entity before fusion, maps naturally onto single-cell multi-omics integration, where dropout, technical noise, and cross-modality misalignment are pervasive. Methods such as scMethyCraft (integrating DNA sequence with methylation), scButterfly, and scBond (translating between transcriptomic and chromatin-accessibility/methylation modalities) face exactly the regime our framework targets. We further note that the repeated-entity caveat we identify for in-batch contrastive losses (the same entity appearing many times per minibatch) recurs there, since single-cell batches contain many cells of the same type or state; the mitigations we describe (anchoring the contrastive signal to the more diverse modality and bounding the alignment weight) are conceptually transferable and may carry over with appropriate batching and masking changes. We see extending intra-entity alignment to these settings as a promising direction.

This work studies multimodal fusion for TCR–peptide binding prediction under noisy structural inputs. We show that naive sequence–structure fusion is unstable relative to strong sequence-only baselines—matching them at full data but degrading toward and below them under protocol shift and limited supervision. Intra-entity contrastive alignment provides an effective mechanism to stabilize multimodal learning by encouraging consistency between sequence and structure representations. The TRACE framework illustrates how such alignment can improve robustness and support the use of structural inductive bias when auxiliary modalities are imperfect.

## Supplementary Material

btag573_Supplementary_Data

## Data Availability

Data are available at https://doi.org/10.6084/m9.figshare.31991007.
