# Supplementary material for: When multimodal fusion fails: contrastive alignment as a necessary stabilizer for TCR–peptide binding prediction
Source: Bioinformatics. 2026 Aug 6;42(8):btag573. doi: 10.1093/bioinformatics/btag573 (PMC13489708; doi:10.1093/bioinformatics/btag573)
Supplement: Supplementary file 1 [file btag573_supplementary_data.pdf]

Supplementary Information

When Multimodal Fusion Fails: Contrastive Alignment as a Necessary Stabilizer for TCR–Peptide Binding Prediction

This document provides complete details of the benchmark, the leakage-controlled evaluation protocol, the model architecture, the training procedure, the full set of per-fold results, the statistical analyses, and the reproduction instructions referenced in the main text. All numbers are produced by the released code under the protocol described in Section S2; no result is reported that is not reproducible from a logged run.

Contents

S1 Benchmark and data **2**
- S1.1 Provenance . . . 2
- S1.2 Negative-generation modes . . . 2
- S1.3 Preprocessing . . . 2
- S1.4 Per-fold statistics . . . 2

S2 Split protocol and leakage controls **3**
- S2.1 Granularity of the split . . . 3
- S2.2 Pair-overlap audit and exclusion . . . 3
- S2.3 Metadata leakage . . . 3
- S2.4 Memorization upper bound . . . 4
- S2.5 Unseen-epitope (strict) setting . . . 4

S3 Model architecture **4**
- S3.1 Sequence tower . . . 4
- S3.2 Graph construction . . . 5
- S3.3 Graph tower . . . 5
- S3.4 Intra-entity contrastive alignment . . . 5
- S3.5 Fusion and binding head . . . 5
- S3.6 Hyperparameters . . . 5

S4 Training and evaluation **5**
- S4.1 Optimization . . . 5
- S4.2 Ensembling and per-epitope normalization . . . 5
- S4.3 Metrics . . . 6
- S4.4 Statistical testing . . . 6

S5 Full results **6**
S5.1 Per-fold, per-method AUROC . . . 6
S5.2 Alignment ablation and significance . . . 6
S5.3 Sensitivity and robustness sweeps . . . 7
S5.4 No structural-prior (PDB) advantage . . . 7

S6 Baselines **7**
S6.1 Protocol-matched baselines (this work) . . . 7
S6.2 External baselines . . . 8

S7 Reproducibility **8**
S7.1 Environment . . . 8
S7.2 Inference wall-clock timing . . . 8
S7.3 Code and data . . . 9
S7.4 Determinism . . . 9

S1 Benchmark and data

S1.1 Provenance

We build on the **TC-hard** benchmark for TCR–peptide binding, which aggregates binding pairs from VDJdb, IEDB, and McPAS-TCR and defines *hard* (epitope-aware) train/test splits. Each example is a triple $(\text{CDR3}\beta, \text{epitope}, y)$ with binary label y. We use the `pep+cdr3b` configuration (TCR β-chain CDR3 + epitope); the benchmark also distributes a `pep+cdr3b+cdr3a+MHC` configuration that additionally carries the paired α-chain and MHC allele, which we discuss as a future extension in the main text.

S1.2 Negative-generation modes

TC-hard provides two distinct negative-construction protocols, both of which we audited:

- `only-sampled-negs` (the random-negative, "RN", setting used in the main text): negatives are synthesized by randomly pairing observed TCRs with epitopes not recorded as their binders.
- `only-neg-assays`: negatives are experimentally determined non-binders from binding assays.

The RN setting is the one against which optimistic-evaluation concerns have been raised in the literature, and is therefore the setting we scrutinise most carefully here.

S1.3 Preprocessing

We map columns `cdr3.beta`→tcrb and `antigen.epitope`→ peptide, replace the non-standard symbol `O` by `X`, and remove a small set of malformed CDR3 entries (five sequences containing internal stop/unknown symbols). TCR and peptide sequences are encoded for the sequence tower with a frozen ESM-2 (650M) model (mean-pooled, 1280-d) and for the graph tower as residue contact graphs (Section S3).

S1.4 Per-fold statistics

Table S1 reports the size, positive rate, and entity counts of each of the five folds.

Table S1: Per-fold dataset statistics (`only-sampled-negs`). Training uses the augmented `pep+cdr3b2` pool; testing uses the held-out `pep+cdr3b` test split. "#epi" and "#TCR" count distinct epitopes and CDR3β sequences.

	Train				Test		
Fold	n	pos.	#epi	#TCR	n	pos.	#epi
0	350,762	0.377	1360	131,821	60,629	0.333	32
1	361,199	0.394	1360	132,026	60,754	0.340	28
2	401,303	0.354	1360	132,026	63,181	0.344	24
3	398,278	0.355	1360	132,024	60,495	0.345	25
4	398,265	0.355	1360	132,025	60,761	0.339	27

S2 Split protocol and leakage controls

This section details, fold by fold, exactly how the data are split and which leakage channels we identified and eliminated. It directly answers the evaluation-rigor questions raised in review.

S2.1 Granularity of the split

The split is performed at the **epitope and pair level**, not at the row level:

- **Epitope-aware.** The TC-hard "hard" design holds out epitopes; the test set contains 24–32 distinct epitopes per fold (Table S1).
- **Pair-disjoint (enforced).** We additionally guarantee that *no* (TCR, epitope) pair present in the test set appears in the training set, by explicitly removing all such rows from training before any model is fit (Section S2.2).
- **Validation by held-out epitopes.** Model selection uses a validation set carved *from training only*, constructed by holding out whole epitopes (peptide-disjoint), with target ratio 0.2 and a fixed seed (42). The test set is never consulted for early stopping or hyperparameter choice.

S2.2 Pair-overlap audit and exclusion

We measured, for each fold, how many test-positive pairs coincide with training-positive pairs in the augmented training pool *before* our exclusion step (Table S2). The overlap is substantial and fold-dependent (50–100%), which is precisely the pair-level leakage that inflates random-negative AUROC. We remove *every* overlapping pair (and any other test pair) from training; all main-text results are obtained after this exclusion.

S2.3 Metadata leakage

We found that several metadata fields distributed with the benchmark *perfectly* encode the label, because they record *how each row was generated* rather than any property of binding. Table S3 shows the cross-tabulation for fold 1: the field `negative.source` takes the value `randomized` for every synthesized negative and is empty for every (database-sourced) positive; `license` and `mhc.source` behave analogously. A one-line rule on `negative.source` yields AUROC ≈ 1.0. **We exclude all such metadata**; only CDR3β and epitope sequence information enters the model.

Table S2: Pair-level overlap between test positives and training positives *before* exclusion. We remove all of these from training; reported results use the pair-disjoint training set.

Fold	test-positive pairs also in train (pos.)	fraction
0	10,088 / 20,177	50.0%
1	20,650 / 20,650	100%
2	21,711 / 21,711	100%
3	19,821 / 20,841	95.1%
4	19,561 / 20,619	94.9%

Table S3: Metadata leakage (fold 1). Each field deterministically separates the labels, because it records the data-generation procedure. All such fields are excluded from the model inputs.

Field	value	label=0	label=1
`negative.source`	`randomized`	40,104	0
	(empty)	0	20,650
`license`	(empty)	40,104	1
	iedb / vdjdb / mcpastcr	0	20,649
`mhc.source`	(empty)	40,104	9,013
	assay / iedb-ligand	0	11,637

S2.4 Memorization upper bound

To verify that a *sequence* model cannot exploit residual pair structure once metadata are removed, we ran a deliberate stress test in which 100% of the test-positive pairs were inserted into the training set (the maximal pair leakage). Even then, test AUROC reached only 0.528, i.e. memorizing the answer pairs does not transfer through sequence features, and the only route to inflated scores is the metadata shortcut of Section S2.3, which we exclude. This bounds the residual leakage risk of our protocol.

S2.5 Unseen-epitope (strict) setting

For completeness we also evaluated the strict *epitope-disjoint* setting (the matched `pep+cdr3b` train/test pair, in which the test epitopes never appear in training). There all methods, including strong cross-attention and interaction-map baselines, operate at or below AUROC 0.55, consistent with the well-documented difficulty of generalizing to unseen epitopes from β-chain sequence alone. We report the seen-epitope, pair-disjoint setting in the main text as the more informative regime, and state this limitation explicitly.

S3 Model architecture

S3.1 Sequence tower

Each entity (TCR, peptide) is embedded by a frozen **ESM-2** (650M parameters, d_s=1280) protein language model, mean-pooled over residues. A two-layer projection MLP maps this to the shared space $\mathbb{R}^D$, D=256. The pLM is not fine-tuned; embeddings are pre-computed once.

S3.2 Graph construction

We predict the 3D fold with ESMFold and represent each residue by its Cα atom. Node features are a 20-dimensional one-hot amino-acid identity. Edges are (i) sequence edges between consecutive residues and (ii) spatial edges between residues with Cα–Cα distance < 8 Å; edges are bidirectional. **No ESMFold per-residue confidence (pLDDT) or coordinate values are used as features**: ESMFold contributes only the binary contact topology. Consequently, low-confidence coordinates can only perturb which contacts exist, a robustness we test directly via edge dropout (Section S5.3).

S3.3 Graph tower

A message-passing GNN processes the residue graph. At layer ℓ,

$$h_i^{(\ell)} = \sigma\Big(W^{(\ell)} h_i^{(\ell-1)} + \sum_{j \in \mathcal{N}(i)} M^{(\ell)} h_j^{(\ell-1)}\Big),$$

followed by global mean pooling and a projection to $\mathbb{R}^D$. We use 2 layers (robustness to 3 layers is reported in Section S5.3) with hidden dimension 256.

S3.4 Intra-entity contrastive alignment

For each entity we align its sequence view $\mathbf{z}^{\text{seq}}$ and graph view $\mathbf{z}^{\text{graph}}$ with a symmetric InfoNCE loss over ℓ_2-normalized embeddings at temperature τ=0.07, computed per entity (TCR and peptide) and averaged. Because the benchmark contains few distinct epitopes, minibatches repeat the same peptide many times; we therefore (i) let the alignment signal be dominated by the near-unique CDR3β side and (ii) keep λ_{align} within the stable range identified in Section S5.3. Training was stable across all seeds under these settings.

S3.5 Fusion and binding head

Within each entity, the two views are fused by concatenation followed by an MLP. Given fused TCR and peptide representations $\mathbf{h}_t, \mathbf{h}_p$, the interaction feature $[\mathbf{h}_t, \mathbf{h}_p, |\mathbf{h}_t - \mathbf{h}_p|, \mathbf{h}_t \odot \mathbf{h}_p]$ is passed to a small MLP that predicts binding.

S3.6 Hyperparameters

S4 Training and evaluation

S4.1 Optimization

All models are trained with AdamW (lr 5×10^{-4}, weight decay 10^{-3}), batch size 128, a one-cycle learning-rate schedule, and class-balanced cross-entropy, with early stopping on validation AUROC (patience 8).

S4.2 Ensembling and per-epitope normalization

For each method and fold we train multiple seeds/configurations and form a validation-selected ensemble by rank-averaging the top models' scores (selection uses validation AUROC only). At test time we additionally apply per-epitope score normalization: scores are z-scored within each

Table S4: Complete hyperparameter settings.

Component	Setting
Sequence tower	ESM-2 650M (1280-d), frozen, mean-pooled
Shared dim D	256
Graph tower	message-passing GNN, 2 layers (tuned $\{2, 3\}$), hidden 256
Pooling	global mean
Alignment	symmetric InfoNCE, $\tau = 0.07$, per-entity
λ_{align}	0.2 (aligned) / 0.0 (ablation)
λ_{bind}	1.0
Class weighting	balanced cross-entropy
Optimizer	AdamW, lr 5×10^{-4}, weight decay 10^{-3}
Schedule	one-cycle
Batch size	128
Early stopping	on validation AUROC, patience 8
Seeds	multiple; final predictions are validation-selected ensembles
Test-time	per-epitope score normalization (uniform across all methods)

test epitope group. This step uses only the epitope grouping (a known input), never labels, and is applied *uniformly to all methods* for fair comparison. It is, however, a *transductive* calibration in that it uses the test-set score distribution within each epitope group, so for completeness we also report results without it: removing per-epitope normalization changes the pooled AUROC only marginally and not in TRACE's favor (matched ensemble 0.568 with vs. 0.570 without), so none of our conclusions depend on this step. A non-transductive alternative is to derive the normalization statistics from the validation/training scores; we expect similar behavior given the small effect size.

S4.3 Metrics

We report AUROC and AUPR, where AUPR is the average-precision estimator (`average_precision_score`); this avoids the optimistic trapezoidal interpolation that can overstate PR-AUC for degenerate predictors.

S4.4 Statistical testing

For the alignment ablation we report (i) a paired t-test and Wilcoxon signed-rank test over the five folds, and (ii) a pooled paired bootstrap over test pairs (2000 resamples) for the AUROC difference. We treat the conservative five-fold test as primary.

S5 Full results

S5.1 Per-fold, per-method AUROC

Table S5 gives the complete per-fold breakdown underlying the controlled comparison in the main text. TRACE (+CLIP) attains the best mean and the best single-fold AUROC; all interaction-aware models improve over sequence-only pooling.

S5.2 Alignment ablation and significance

The per-fold CLIP ablation is in Table S5 (last two rows). The full-data difference is +0.013 mean AUROC (+CLIP $\geq$ no-CLIP on 4/5 folds). The conservative five-fold paired test does not

Table S5: Per-fold test AUROC for every method under the identical controlled protocol (pair-disjoint, metadata-controlled, validation-only-selected; validation-selected ensemble with per-epitope normalization).

Method	f0	f1	f2	f3	f4	mean
Seq-only (pooled ESM)	0.578	0.532	0.576	0.494	0.556	0.547
Cross-attention (seq)	0.591	0.565	0.586	0.507	0.562	0.562
Interaction map (Imrex-style)	0.600	0.572	0.573	0.489	0.564	0.560
TRACE (no CLIP)	0.572	0.573	0.582	0.535	0.561	0.565
**TRACE (+CLIP)**	0.601	0.589	0.606	0.524	0.570	**0.578**

reach significance (paired t-test $p = 0.12$; Wilcoxon $p = 0.19$; $n = 5$), reflecting limited fold count; a pooled paired bootstrap places the difference reliably above zero (mean +0.0136, 95% CI $[+0.012, +0.016]$). The benefit becomes large and unambiguous under data scarcity (Section S5.3). We therefore characterize intra-entity alignment as a stabilizer with a modest full-data benefit.

S5.3 Sensitivity and robustness sweeps

Tables S6–S7 report the complete sweeps (5-fold mean). Two findings: (i) the alignment weight is not a sensitive hyperparameter (all $\lambda > 0$ modestly improve over $\lambda = 0$ and are stable across the range); (ii) the model is robust to substantial graph-contact noise (edge dropout), and under data scarcity the non-aligned model collapses toward chance while alignment preserves learning (Table S7).

Table S6: Sensitivity to the alignment weight λ_{align} (5-fold mean±std). Every non-zero weight improves over no alignment; the effect is modest and stable.

λ_{align}	AUROC	Δ vs. no align
0 (no alignment)	0.559 ± 0.015	0.000
0.05	0.569 ± 0.025	+0.010
0.1	0.565 ± 0.025	+0.006
0.2 (used)	0.566 ± 0.029	+0.007
0.5	0.564 ± 0.028	+0.005

S5.4 No structural-prior (PDB) advantage

To test whether ESMFold's PDB training set grants "popular" epitopes privileged contact graphs, we examined per-epitope test AUROC. It has essentially zero correlation with epitope popularity (Spearman $\rho = 0.008$, $p = 0.96$), and canonical PDB-represented epitopes show no significant advantage over the remainder (0.599 vs 0.585; t-test $p = 0.81$). Combined with the identity-only node features, this bounds the ESMFold/PDB leakage channel.

S6 Baselines

S6.1 Protocol-matched baselines (this work)

All comparison methods are trained and evaluated under the identical controlled protocol (pair-disjoint, metadata-controlled, validation-only-selected):

Table S7: Robustness sweeps (5-fold mean). *Top:* edge dropout (structural-contact noise): performance is stable, showing the model is not brittle to contact quality. *Bottom:* positive-label downsampling (data scarcity): without alignment the model collapses toward chance; alignment preserves learning, and the gap grows as labels shrink.

Setting	w/o Align	w/ Align	Δ
Edge dropout (structural-contact noise)			
0.0	0.5590	0.5664	+0.0074
0.1	0.5669	0.5642	−0.0027
0.2	0.5609	0.5660	+0.0051
0.3	0.5656	0.5637	−0.0019
0.4	0.5630	0.5596	−0.0034
Positive-label fraction (data scarcity)			
0.1	0.4953	0.5430	+0.0477
0.2	0.4993	0.5538	+0.0545
0.5	0.5443	0.5624	+0.0180
1.0	0.5590	0.5664	+0.0074

- **Seq-only**: frozen ESM-2 embeddings of TCR and peptide, combined as $[\mathbf{t}, \mathbf{p}, |\mathbf{t} - \mathbf{p}|, \mathbf{t} \odot \mathbf{p}]$ and passed to an MLP.
- **Cross-attention (sequence)**: a joint Transformer over the concatenated CDR3β and epitope residues with full pairwise self-attention.
- **Interaction map (Imrex-style)**: a 2D CNN over a CDR3β×epitope residue interaction map built from per-residue physicochemical (Atchley) features.
- **TRACE (no CLIP / +CLIP)**: the proposed dual-tower model with the alignment weight set to 0 or 0.2.

S6.2 External baselines

The external numbers tabulated for context in the main text are taken from the respective original publications. They were obtained on heterogeneous datasets, splits, and negative-sampling protocols, and in several cases without pair- or metadata-leakage control; they are therefore *not* directly comparable to the protocol-matched results and are included only to situate the work.

S7 Reproducibility

S7.1 Environment

Experiments were run with PyTorch on NVIDIA A100 GPUs. With frozen ESM-2 features the trainable model has a few million parameters; a single fold trains in minutes, and per-pair inference is the sequence-only cost plus one GNN forward pass.

S7.2 Inference wall-clock timing

We measured wall-clock inference latency on a single NVIDIA A100 (fp32, eval mode, ESM-2 features precomputed and excluded as a one-off cost shared by all ESM-based methods), timing only the downstream model forward pass over real test-fold pairs (30 warm-up iterations, 200 timed

iterations, CUDA-synchronized). Table S8 reports per-pair latency (ms/pair) across batch sizes for the sequence-only baseline, TRACE (ESM + 2-layer GNN + CLIP, the paper configuration: D=256, graph hidden 256, 2 layers), and a residue-level cross-attention baseline at two widths (d=128 and d=256, 4 layers, 4 heads). The added graph tower raises per-pair cost by roughly 7–9× over the sequence-only baseline but keeps inference well under a millisecond per pair. Relative to cross-attention the comparison is batch-size dependent: at single-pair latency the two are comparable (≈1.7–1.9 ms/pair, where kernel-launch overhead dominates for both), whereas in the batched high-throughput regime relevant for large-scale screening TRACE is ≈2–4× faster per pair (at batch 512, 0.005 ms/pair, i.e. $\approx 1.9 \times 10^5$ pairs/s, versus 0.010–0.024 ms/pair for cross-attention), because the residue graph is built once per unique sequence rather than per pair and the GNN avoids the quadratic CDR3β×epitope attention map.

Table S8: Measured inference latency (ms per pair) on one A100, fp32, eval mode, ESM-2 features precomputed (one-off, excluded). Trainable-parameter counts: seq-only 0.92M, TRACE 1.86M, cross-attention d=128 1.79M, d=256 4.23M. Lower is faster.

Batch size	Seq-only	TRACE	Cross-attn (d=128)	Cross-attn (d=256)
1	0.2768	1.9009	1.7114	1.7480
32	0.0092	0.0620	0.0545	0.0561
128	0.0023	0.0157	0.0136	0.0292
512	0.0006	0.0053	0.0099	0.0238

S7.3 Code and data

Code is available at `https://github.com/MineSelf2016/TRACE` and archived at `https://doi.org/10.5281/zenodo.20635593`; the processed data and splits are available at `https://doi.org/10.6084/m9.figshare.31991007`. The repository includes the data-loading and split-construction scripts (negative-mode selection, peptide-disjoint validation construction with seed 42, and the pair-exclusion step), the model definitions (sequence tower, graph tower, alignment loss, fusion head), the training harness, and the collection/statistics scripts used to produce every table in this document.

S7.4 Determinism

All random seeds (data splitting, model initialization, sampling) are fixed and logged in each run's metadata file, so every reported number can be regenerated from the corresponding command line.
